# Diagnostic utility of apparent diffusion coefficient in preoperative assessment of endometrial cancer: are we ready for the 2023 FIGO staging?

**DOI:** 10.1186/s12880-024-01391-5

**Published:** 2024-08-28

**Authors:** Gehad A. Saleh, Rasha Abdelrazek, Amany Hassan, Omar Hamdy, Mohammed Salah Ibrahim Tantawy

**Affiliations:** 1 Diagnostic Radiology department, Faculty of Medicine, Mansoura University, Mansoura, Egypt; 2 Pathology Department, Faculty of Medicine, Mansoura University, Mansoura, Egypt; 3 Surgical oncology department, Oncology center, Mansoura University, Mansoura, Egypt

**Keywords:** Endometrial cancer, Myometrial invasion, Diffusion weighted MRI, Apparent diffusion coefficient

## Abstract

**Background:**

Although endometrial cancer (EC) is staged surgically, magnetic resonance imaging (MRI) plays a critical role in assessing and selecting the most appropriate treatment planning. We aimed to assess the diagnostic performance of quantitative analysis of diffusion-weighted imaging (DWI) in preoperative assessment of EC.

**Methods:**

Prospective analysis was done for sixty-eight patients with pathology-proven endometrial cancer who underwent MRI and DWI. Apparent diffusion coefficient (ADC) values were measured by two independent radiologists and compared with the postoperative pathological results.

**Results:**

There was excellent inter-observer reliability in measuring ADCmean values. There were statistically significant lower ADCmean values in patients with deep myometrial invasion (MI), cervical stromal invasion (CSI), type II EC, and lympho-vascular space involvement (LVSI) (AUC = 0.717, 0.816, 0.999, and 0.735 respectively) with optimal cut-off values of ≤ 0.84, ≤ 0.84, ≤ 0.78 and ≤ 0.82 mm^2^/s respectively. Also, there was a statistically significant negative correlation between ADC values and the updated 2023 FIGO stage and tumor grade (strong association), and the 2009 FIGO stage (medium association).

**Conclusions:**

The preoperative ADCmean values of EC were significantly correlated with main prognostic factors including depth of MI, CSI, EC type, grade, nodal involvement, and LVSI.

**Supplementary Information:**

The online version contains supplementary material available at 10.1186/s12880-024-01391-5.

## Background

Endometrial cancer (EC) is the most common gynecologic cancer in developed countries with a rising incidence globally [1]. EC is staged surgically by using the International Federation of Gynecology and Obstetrics (FIGO) classification system which was recently updated in 2023 [2] however, magnetic resonance imaging (MRI) plays a critical role in assessment and selecting the most appropriate treatment planning. The surgical staging procedure is total hysterectomy, bilateral salpingo-oophorectomy, assessment of lymph nodes (LNs), and peritoneum [3, 4]. Recently, lymphadenectomy can be evaded in patients with no detected high-risk features at imaging, including patients with less than 50% myometrial invasion (MI), tumors less than 2 cm, and tumor grade 1 or 2 [5].

Histological type is also an imperative prognostic predictor, EC is categorized based on the 5th edition of WHO Classification of Female Genital Tumors to variable histological types including endometrioid carcinoma of low grade (grades 1 and 2) or high grade (grade 3), clear cell carcinoma, serous carcinoma, undifferentiated carcinoma, carcinosarcoma, mixed carcinoma, and other rare types [6]. EC is classified into two main types; type I (non-aggressive) EC includes low-grade endometroid carcinoma while type II (aggressive) EC includes all other histological types, the latter frequently presented with advanced disease and had a poor prognosis [3, 5].

MRI is the best imaging tool for the assessment of key prognostic factors including depth of MI, cervical stromal invasion (CSI), nodal involvement, and extrauterine spread owing to its excellent soft tissue resolution [7, 8]. European Society of Urogenital Radiology considered MRI as the standard diagnostic tool in the pre-treatment setting of EC as it permits risk stratification in low- and high-risk groups as a road map for treatment plans [5, 9]. However, accurate detection of MI depth in conventional MRI may be challenging due to coexisting adenomyosis, leiomyomas, and myometrium compression by large tumors [10]. When CSI is present and the histology at biopsy is not typical, it is sometimes difficult to distinguish whether the origin is from the endometrium or cervix [11]. Utilizing functional MRI techniques such as Diffusion-weighted imaging (DWI) and dynamic contrast-enhanced MRI (DCE-MRI) enhances diagnostic accuracy in such instances [12, 13].

DWI permits qualitative assessment of the tissue microstructure based on its sensitivity to water molecular motion [14], it also provides quantitative assessment through the apparent diffusion coefficient (ADC) which reflects the tissue cellularity [15, 16].

Previous studies have discussed the added value of ADC in the detection of chief prognostic factors in EC patients with variable results concerning its reliability and need for further validation [8, 17, 18]. This study aimed to evaluate the inter-observer reliability in measuring ADC mean values for preoperative assessment and risk stratification of EC. Also, to evaluate the correlation between ADC values and the FIGO stage of EC.

## Methods

### Study population

The local institutional review board approved this prospective study and informed consent for medical records was obtained after patient agreement. From February 2022 to May 2023, eighty-three patients with abnormal uterine bleeding and suspected EC at dilatation and curettage underwent pelvic MRI and DWI and were initially enrolled. Fifteen patients were excluded; 11 received neoadjuvant therapy, two had endometrial stromal sarcoma and the other two underwent surgery at other institutions with missed postoperative pathological results. The final study cohort consisted of 68 consecutive patients with pathology-proven EC.

### MR imaging technique

Pelvic MRI and DWI were performed within 2–4 weeks before surgery on the same 1.5-T MR imaging scanner (Philips Ingenia, Netherlands) using pelvic phased-array surface coils. Patients fasted for 4–6 h before the examination and were injected 20 mg of Butylscopolamine bromide to reduce peristalsis-related artifacts. Pelvic MR examination comprised the following sequences: axial T1-weighted image (T1WI) (TR/TE, 400–600/10–14 ms), high resolution sagittal and axial oblique (perpendicular to the endometrial cavity) T2WI (TR/TE, 4000–6000/100-110ms), slice thickness/ interslice gap, 4 mm/1 mm; matrix, 320 × 320). MRI technique also included axial T2WI of the abdomen (from renal hila to symphysis pubis) to assess nodal and bony metastases (slice thickness/interslice gap, 6 mm/1 mm).

DWI was performed before the contrast material injection using an axial fat-suppressed single-shot echo-planar imaging with variable b values (b = 0,500,1000 s/mm^2^). Scanning parameters were as follows: TR/TE = 7000/77ms, FOV = 240 × 220, matrix = 128 × 128, slice thickness = 4 mm, and slice gap = 1 mm. post-contrast axial, sagittal, and coronal T1WI (TR/ TE of 800/15 ms) were obtained 2 minutes and 30 s after intravenous injection of 0.1 mL/kg of Gadopentetate dimeglumine at a rate of 2 mL/s.

### MR image analysis

The images were transferred to a workstation (extended MR Workspace 2.6.3.5, Philips Medical System). Image analysis was performed by two radiologists (one with five years of experience and the other with thirteen years of experience in pelvic MRI). Both radiologists were blinded to the tumor type and grade.

#### Conventional MRI analysis

The following MRI features were documented for each patient by the two radiologists in consensus; Short MR axis tumor size (mm) on sagittal T2WI, depth of MI; considering deep MI when the tumor involved greater than 50% of the myometrium, CSI; defined as disruption of normal cervical stromal T2 hypointensity and enhancement [5], ovarian or vaginal involvement, nodal involvement, extrauterine spread, presence of ascites, peritoneal deposits, and distant metastasis.

#### DWI analysis

Matched ADC maps were applicable using a Phillips Advantage Windows workstation with functional tool software.

- Qualitative DWI analysis: DWI was first evaluated qualitatively by visual assessment of the signal intensity of the endometrial mass, a hyperintense signal at a high b-value (1000s/mm^2^) with a hypointense signal on the corresponding ADC map was considered restricted diffusion.
- Quantitative DWI analyses: Quantitative analyses were performed by the two radiologists independently. Each radiologist measured the mean ADC (ADCmean) values by manually drawing a circular 2D region of interest (ROI) on the axial ADC map encompassing the darkest part of the endometrial mass with references to T2 and post-contrast images to evade necrotic areas. ROIs varied in size from 1.1 to 3.5 cm^2^. The ADCmean values were measured three times, and the measurements were averaged.

### Histopathological analysis

The following histopathological data were acquired after surgery: histological type, tumor grade, depth of MI, presence of CSI, ovarian involvement, lympho-vascular space involvement (LVSI), and nodal status, the FIGO stage was assigned according to the 2009 FIGO staging. Recently, the updated 2023 FIGO staging of EC was released to include more histopathological and molecular details [19]. All cases were retrospectively assessed by a pathologist with 13 years’ experience in gynecological cancers and updated 2023 FIGO stages (which were released during the preparation of the manuscript) were also assigned and compared with the ADC measurements.

### Statistical analysis

Data were analyzed using IBM-SPSS software (version 27, 2020) and MedCalc Statistical Software (version 18.9.1). Qualitative data is N (%) compared by chi-square test. Quantitative data were initially tested for normality using Shapiro-Wilk’s test, with data being normally distributed if *p* > 0.050. Cohen’s κ which was run to determine if there was agreement between radiological techniques and pathological results. The diagnostic performance of quantitative ADC measurements was assessed by ROC curve analysis to find the cutoff value of ADC to discriminate between EC types. One-way ANOVA test compared ADC measurements between the three EC grades. The Spearman’s correlation test was used to determine whether there is a linear relationship/association between two non-normally distributed quantitative data. The intraclass correlation coefficient (ICC) and Bland and Altman plot were used to judge the agreement of ADC measurements between the two raters. Cohen’s weighted kappa was run to test the agreement between two FIGO staging systems. For any of the used tests, results were considered statistically significant if the p-value ≤ 0.050.

## Results

### Patients’ characteristics

Among the sixty-eight included cases the mean age (years) ± SD was 60.5 ± 7.8 years, 85.3% had endometroid carcinoma and 70.6% had grade 1 or 2 EC. Type I EC was reported in 70.6% of cases with no detected statistically significant difference in age between EC types. Stage 1 A was the most common FIGO stage according to both the 2009 FIGO and updated 2023 FIGO staging systems (54.4% and 47% respectively). Further patient characteristics are shown in (Table 1).

**Table 1:** Radiological and pathological characteristics

Patient’s characteristic	*N*	%
Myometrial invasion (DWI) > 50%	31	45.6
Myometrial invasion (CE) > 50%	34	50
Myometrial invasion (Pathology) > 50%	28	41.2
LVSI (lymphovascular space involvement)	29	42.6
Cervical stromal invasion (CSI)	10	14.7
Histological type		
Type 1	48	70.6
Type 2	20	29.4
Tumor grade		
Grade 1	14	20.6
Grade 2	34	50
Grade 3	20	29.4
Pathological subtypes:		
Carcinosarcoma	3	4.4
Clear cell carcinoma	1	1.5
Serous carcinoma	5	7.4
Undifferentiated carcinoma	1	1.5
Endometroid carcinoma	58	85.3
FIGO stage		
IA	37	54.4
IB	17	25.0
II	2	2.9
IIIA	2	2.9
IIIC	10	14.7
Updated 2023 FIGO stage		
IA	32	47
IB	12	17.6
IIA	1	1.5
IIC	12	17.6
IIIA	1	1.5
IIIB	1	1.5
IIIC	9	13.2

### MRI analysis

There was very good agreement between DWI and CE-MRI (κ = 0.853, *p* < 0.001) in detecting the depth of MI. The two techniques agreed on thirty-three cases exhibiting superficial MI and thirty cases exhibiting deep MI. However, CE-MRI rated four cases with deep MI when DWI rated them with superficial MI, and CE rated only one case as superficial MI when DWI rated this case as exhibiting deep MI. There was very good agreement between the two techniques, κ = 0.853, *p* < 0.001. Furthermore, there was a superior diagnostic accuracy of DWI in detecting the depth of MI compared to CE-MRI, there was very good agreement between DWI and pathological degree of MI, κ = 0.851, *p* < 0.001 while CE-MRI revealed good agreement with the pathological results, κ = 0.706, *p* < 0.001 (supplementary Fig. 1). Also, there was a statistically significant association between LVSI and Short MR axis tumor size on sagittal T2WI, χ2 (1) = 19.403, *p* < 0.001.

**Fig. 1:**
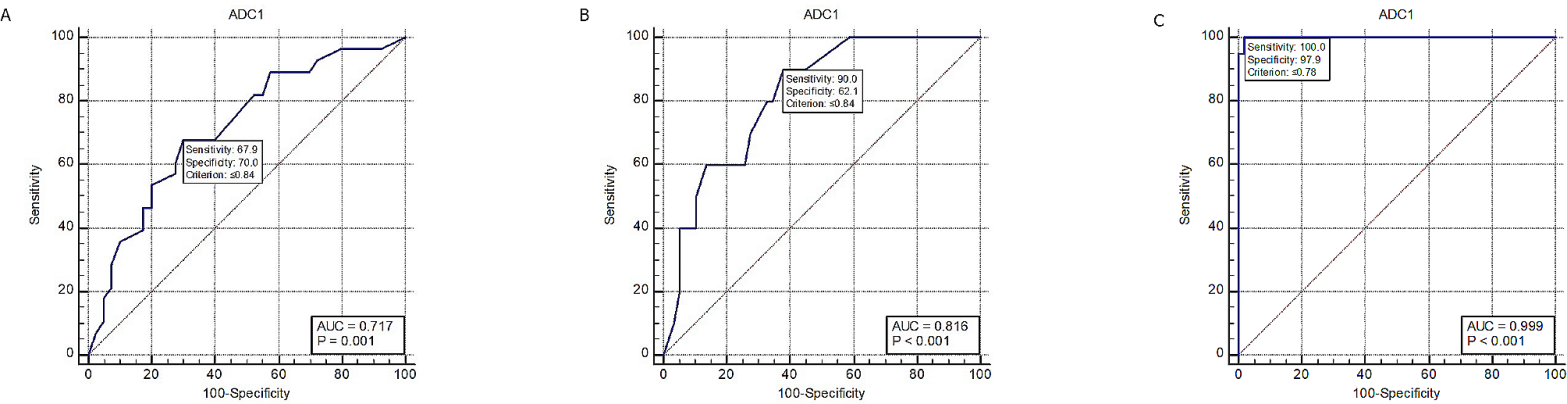
ROC curves for diagnostic performance of ADCmean in the prediction of depth of MI, CSI, and EC type (A, B & C respectively)

There was a statistically significant difference in ADCmean values in correlation with the depth of MI and presence of CSI, with lower ADCmean values in patients with deep MI and CSI. The ROC curve analysis revealed that the ADCmean at a cut-off value of ≤ 0.84 had an acceptable and excellent discrimination of patients with deep MI and CSI versus those with superficial MI and absent CSI respectively (AUC = 0.717 and 0.816; *p* < 0.001 and *p* < 0.001 respectively) (Fig. 1a & b).

There were also statistically significant lower ADCmean values in patients with type II versus those with type I EC. ROC curve analysis revealed that ADCmean at a cut-off value of ≤ 0.78 mm^2^/s had outstanding discrimination with 100% sensitivity and 97.9% specificity to discriminate type II from type I EC. (AUC = 0.999; *p* < 0.001) (Fig. 1c).

**Fig. 2:**
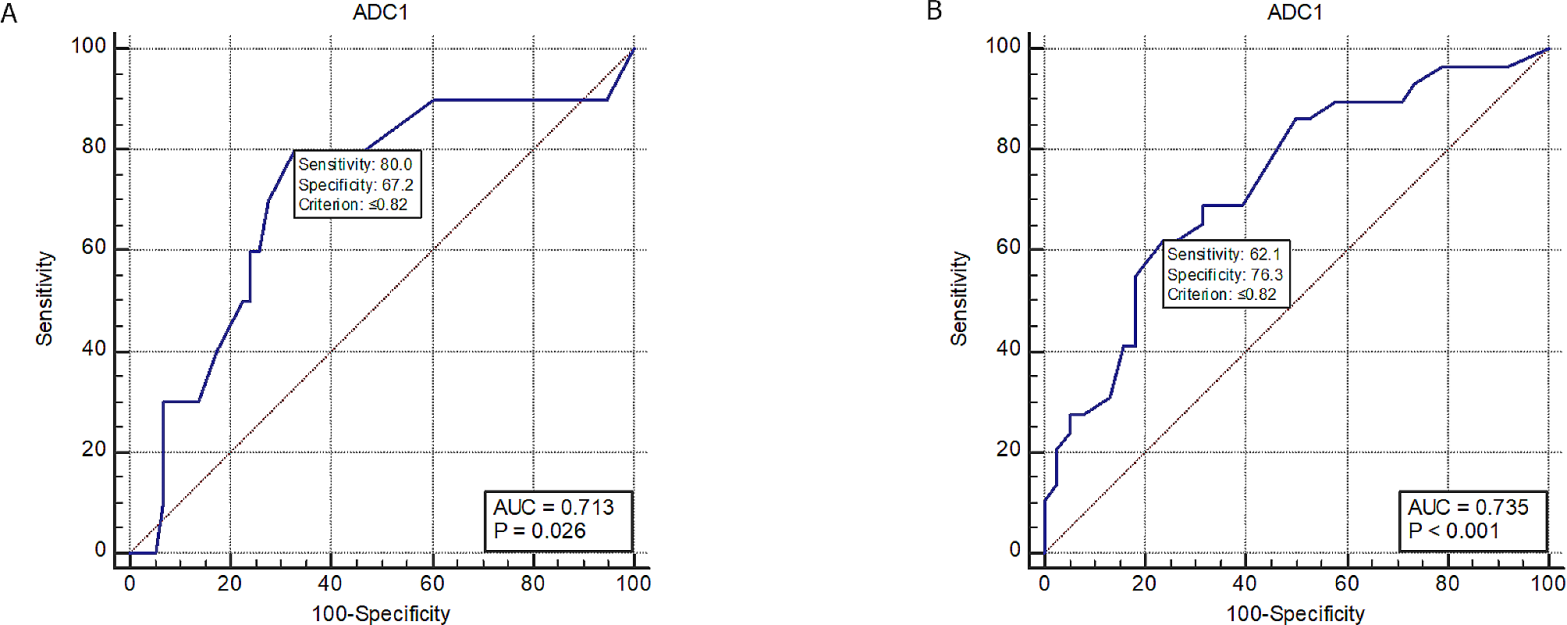
ROC curves for diagnostic performance of ADCmean in prediction of nodal involvement and LVSI (A&B respectively)

There were statistically significant lower ADCmean values in patients with nodal involvement and LVSI. ROC curve analysis revealed that ADCmean at a cut-off value of ≤ 0.82 had acceptable discrimination of patients with nodal involvement and LVSI (AUC = 0.713 and 0 = 735; *p* = 0.026 and *p* < 0.001 respectively) (Fig. 2).

Also, there was a statistically significant difference in ADC values regarding tumor grades with statistically significantly higher ADC values in grade 1 > grade 2 > grade 3 (*p*-values < 0.001) (Table 2).

**Table 2:** ADC values in tumor grades

Grade	*n*	Mean	SD	F [2, 65]	*p*-value	P1	P2	P3
Grade 1	14	1.0007	0.076	83.977	**< 0.001**	**< 0.001**	**< 0.001**	**< 0.001**
Grade 2	34	0.9053	0.091					
Grade 3	20	0.6770	0.049					

Notes: SD = standard deviation. The test of significance is the One-Way ANOVA test. Results of Tukey HSD tests were presented as P1 (significant difference between grade 1 vs. grade 2), P2 (significant difference between grade 1 vs. grade 3), and P3 (significant difference between grade 2 vs. grade 3)

**Fig. 3:**
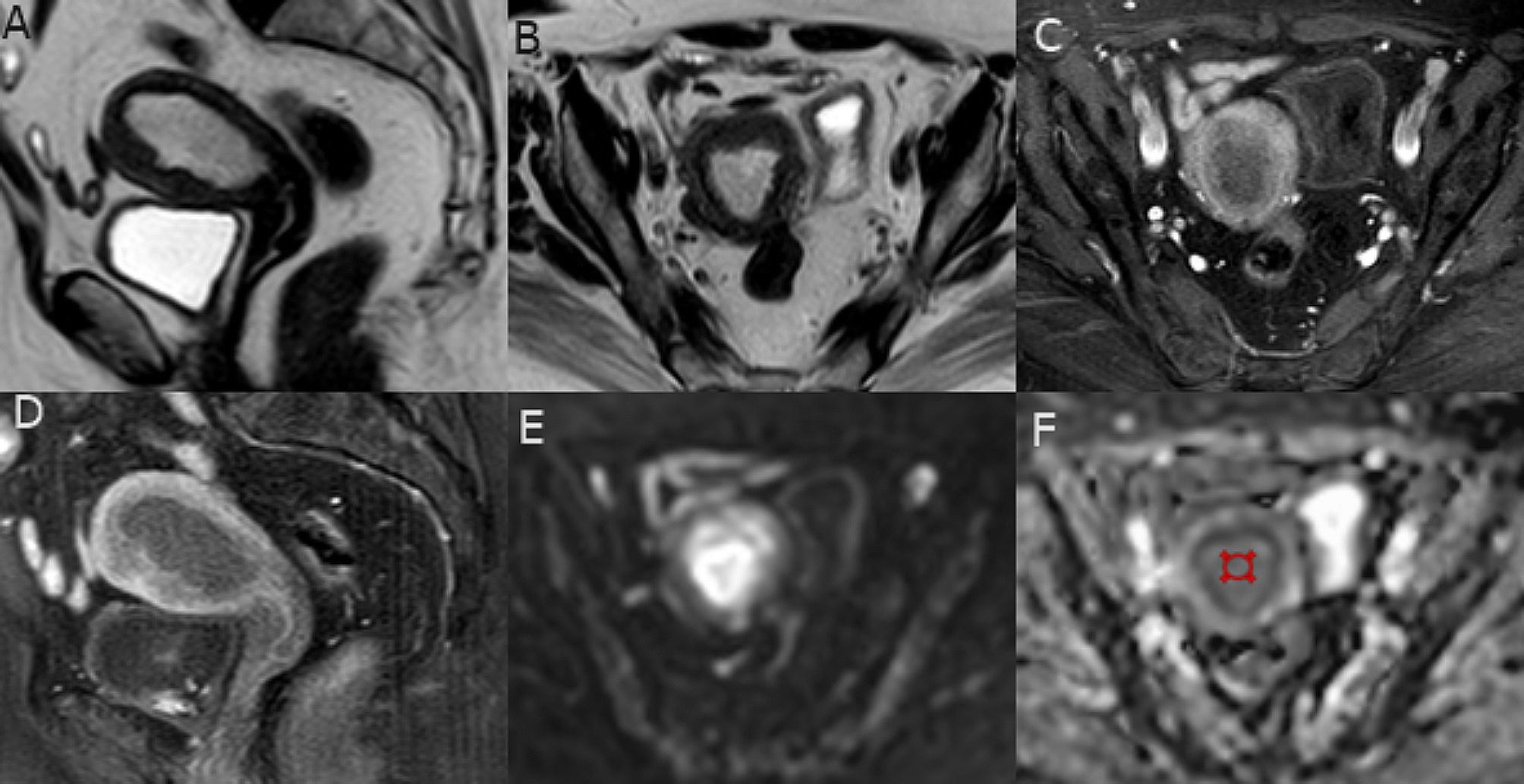
A patient with grade 2 endometroid carcinoma, no LVSI (FIGO stage IA). (**A** and **B**) Sagittal and axial oblique T2-WI respectively showed the endometrial mass of intermediate to high signal intensity with focally interrupted hypointense junctional zone denoting superficial MI, no detected CSI nor pathological pelvic LNs. (**C** & **D**) Axial oblique and sagittal contrast-enhanced T1-WI revealed interrupted sub-endometrial enhancement with hypo-enhancement of the endometrial mass. (**E** & **F**) Axial DWI at b = 1000 and ADC map respectively revealed diffusion restriction of the endometrial mass with ADCmean value = 1.02 × 10 –3 mm/sec

**Fig. 4:**
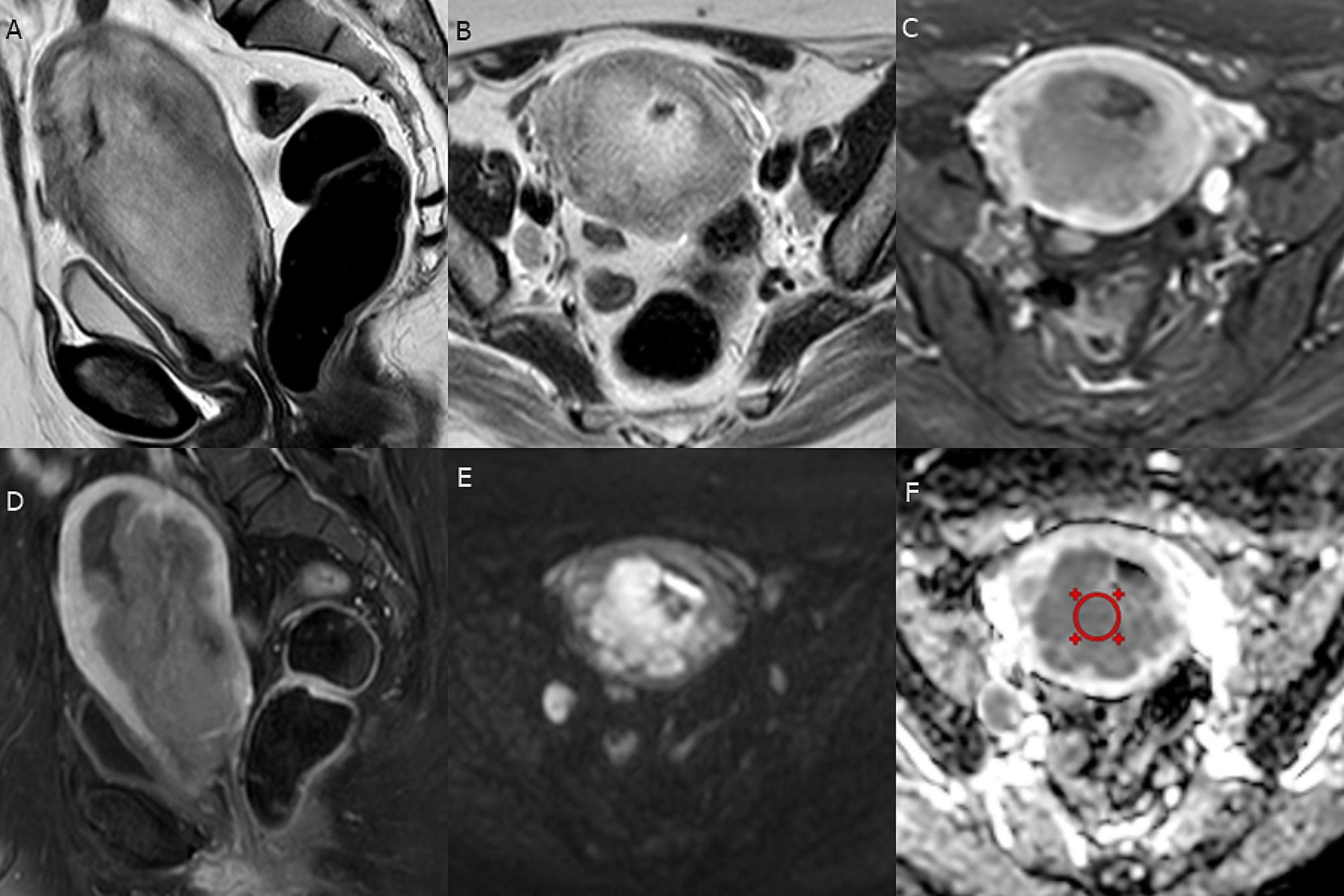
A patient with endometrial serous carcinoma and LVSI (FIGO stage III C1). (**A** and **B**) Sagittal and axial oblique T2-WI respectively showed the large endometrial mass of heterogeneous intermediate signal intensity with lost hypointense junctional zone and deep MI, interrupted hypointense cervical stroma denoting CSI, and globular suspicious right internal iliac LN. (**C** & **D**) Axial oblique and sagittal contrast-enhanced T1-WI revealed a heterogeneous enhancement of the endometrial mass, deep MI, CSI, and heterogeneously enhanced necrotic right internal iliac LN. (**E** & **F**) Axial DWI at b = 1000 and ADC map respectively revealed marked diffusion restriction of the endometrial mass and right iliac LN with ADCmean value = 0.77 × 10 –3 mm/sec

Overall, there was a statistically significant negative correlation between ADC values and updated 2023 FIGO stage and tumor grade (strong and very strong strength of association respectively), and FIGO stage and short MR axis tumor size (moderate strength of association) (Table 3). Demonstrative cases are shown in (Figs. 3 and 4).

**Table 3:** Correlations of ADC values

Characteristic	Correlation coefficient	*P* value
FIGO stage	− 0.488	**< 0.001**
2023 FIGO stage	− 0.705	**< 0.001**
Short MR axis tumor size (mm)	− 0.489	**< 0.001**
Tumor grade	− 0.816	**< 0.001**

Notes: The test of significance is Spearman’s correlation test

### Inter-observer reliability

**Fig. 5:**
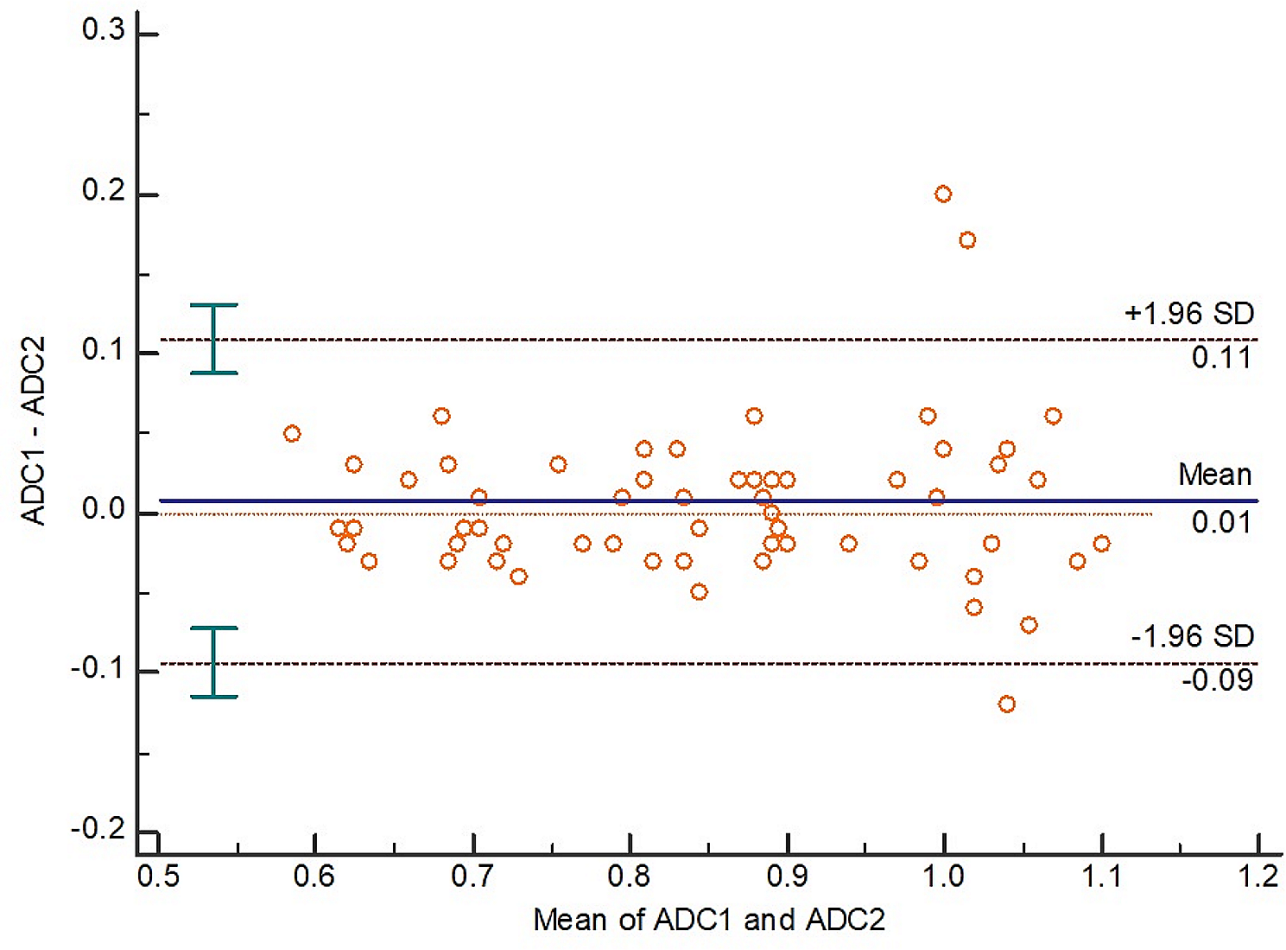
Bland-Altman plot for inter-observer reliability of ADCmean measurements

There was excellent reliability (absolute agreement) between the two observers in measuring ADCmean values with ICC = 0.934 and 95% CI = 0.895–0.959, (Fig. 5).

### Agreement between 2009 FIGO and updated 2023 FIGO staging systems

There was strong agreement between the two FIGO staging systems (weighted kappa [95% CI] = 0.619 [0.524-0.714], *p* < 0.001). The 2009 FIGO assigned thirty-seven cases as stage IA, thirty-two agreed with the 2023 FIGO while the other five cases were assigned as stage IIC. Furthermore, the updated FIGO assigned thirty-two cases as stage IA, and all of them agreed with the 2009 FIGO. The 2009 FIGO diagnosed seventeen cases as stage IB, twelve cases agreed with the 2023 FIGO while five cases were diagnosed as stage IIC by updated FIGO. On the other hand, the updated FIGO diagnosed ten cases as stage IB, and all of them agreed with the 2009 FIGO.

The 2009 FIGO assigned two cases as stage II, one case was diagnosed as stage IIA by the updated FIGO, and the other one was assigned as IIC. On the other hand, the updated FIGO diagnosed one case as stage IIA which was assigned as II by the 2009 FIGO. Also, the new FIGO diagnosed twelve cases as IIC, which were diagnosed as IA (five cases), IB (five cases), II (one case), and IIIC (one case).

**Fig. 6:**
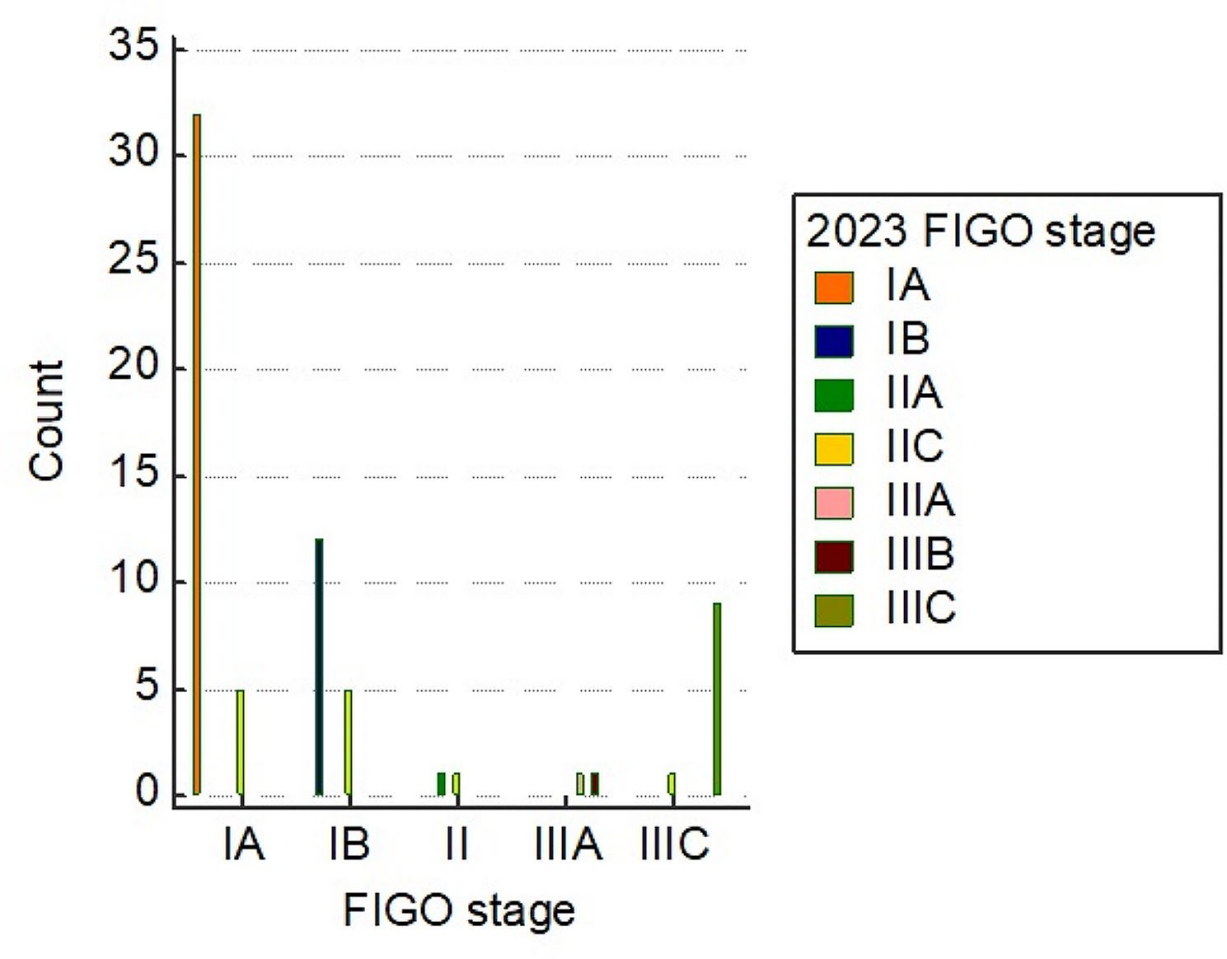
Bar chart for the agreement between 2009 FIGO and updated 2023 FIGO staging systems

The 2009 FIGO diagnosed two cases as stage IIIA, which were diagnosed by the 2023 FIGO as IIIA (one case) and IIIB (one case). While updated FIGO diagnosed one case as stage IIIA, which agreed with 2009 FIGO. Furthermore, the 2009 FIGO identified ten cases as stage IIIC, which were diagnosed by the updated FIGO as IIC (one case) and IIIC (9 cases). On the other hand, a new FIGO diagnosed one case as stage IIIB, which was diagnosed with 2009 FIGO as IIIA, and nine cases as IIIC, all agreed with the 2009 FIGO (Table 4, Fig. 6).

**Table 4:** Agreement between 2009 FIGO and updated 2023 FIGO staging systems

Updated 2023 FIGO stage	FIGO stage					
	IA	IB	II	IIIA	IIIC	Total
IA	32	0	0	0	0	32
IB	0	12	0	0	0	12
IIA	0	0	1	0	0	1
IIC	5	5	1	0	1	12
IIIA	0	0	0	1	0	1
IIIB	0	0	0	1	0	1
IIIC	0	0	0	0	9	9
Total	37	17	2	2	10	68

Notes: Data is the absolute frequency (N)

## Discussion

It is critical to differentiate superficial from deep MI, as the latter is associated with a high risk for LVSI, which relates directly to tumor grade, nodal metastases, and recurrence [4]. Our results revealed statistically significant lower ADCmean values in patients with deep MI versus those with superficial MI. That was in line with another prospective study which revealed that the ADCmean values were significantly lower in tumors with deep MI and were also considered unfavourable prognostic factors [20]. Similarly, a recent retrospective study concluded that low ADC values were associated with deep MI [21]. Unlike our results, inter-observer reliability was not accomplished in their studies. Our study also reported excellent interobserver reliability in measuring ADCmean values, which was in line with a previous prospective study conducted on 53 EC patients [8]. A previous retrospective study revealed that deep MI is significantly associated with high-grade EC, however, they concluded that ADC histogram analysis was not beneficial for envisaging EC-grade [22].

In addition, our results revealed better diagnostic accuracy of DWI than CE-MRI to discriminate deep from superficial MI. That was in line previous retrospective study revealed superior diagnostic accuracy of DWI in detecting MI compared to CE-MRI with no statistically significant difference. Unlike our results, the added value of ADC was not discussed in their study [23]. Similarly, a previous meta-analysis study concluded that DWI-T2WI predicts the depth of MI better than DCE-MRI and considered DWI as an alternative for DCE-MRI in preoperative staging of EC [24].

Regarding CSI, our results revealed statistically significant lower ADCmean values in patients with CSI. Similarly, a previous prospective study on 47 EC patients revealed lower ADC values in patients with CSI [25]. However, another study concluded no significant difference in ADC values among patients without or with CSI [26].

Furthermore, our results exhibited statistically significant lower ADCmean values in patients with type II versus those with type I with an outstanding discrimination at a cut-off value of ≤ 0.78. Comparable results were concluded by a previous retrospective study with ADCmean optimal cut-off value of 0.75 for discrimination between the two types [18].

There was a statistically significant negative correlation between ADCmean values and EC grades, which was in line with the previous retrospective studies on EC [27–29]. Similarly, a recent prospective study on 44 EC patients revealed statistically lower ADC values of grade 3 compared to Grade 1–2 EC [17]. A previous retrospective study reported significantly lower ADC values of EC than that of benign endometrial lesions with no statistically significant difference in ADC values between EC grades, thus assuming the small number of included EC cases in their study (23 EC patients) [30]. Previous retrospective studies concluded significant differences in ADC values among three risk categories of EC with lower values in the intermediate and high-risk groups compared to the low-risk group [31, 32]. This can help the surgeons plan an appropriate surgical decision based on the imaging findings.

Also, our results revealed statistically significant lower ADCmean values in patients with nodal involvement and LVSI. Similarly, a recent retrospective study revealed an inverse correlation of the ADC value of EC primary lesion and pelvic LN metastasis with an optimal ADC cut-off value of 0.908 [33]. Our result revealed a statistically significant association between LVSI and Short axis > 24 mm on sagittal T2WI, which was in line with the previous retrospective study, but there were no significant differences in ADC values among cases with or without LVSI [34]. The available data relating ADC values and LVSI is limited. However, few previous studies revealed no significant difference in ADC values among cases with LVSI or nodal involvement [17, 29].

Stage 1 A was the most common (54.4%) FIGO stage in our study, which was in line with the previous retrospective study [23]. Also, our results revealed a statistically significant negative correlation between ADCmean values and the FIGO stage. Similarly, a previous recent study reported a significant difference in ADC values when comparing Stage IA and Stage III EC [17].

We aimed to explore the value of the recently published 2023 FIGO staging system, our results revealed was strong agreement between the two FIGO staging systems. Furthermore, there was a statistically significant negative correlation between ADC values with the 2009 FIGO stage and the updated 2023 FIGO stage with medium and strong strength of association, respectively. Further prospective studies are recommended to confirm the accuracy of ADC in correlation with the updated FIGO stage.

This study has a few limitations: First, the single-center study design. Second, a small number of the included non-endometroid EC. Third, the included patients are those who underwent surgical intervention only in an oncology referral centre so patients with abnormal uterine bleeding of non-oncological etiology were excluded. Further, multicentre studies are recommended to judge the impact of ADC measurements on preoperative treatment plans. Recent studies have assessed the effectiveness of MRI-based radiomics in diagnosing and staging EC [35–38]. Future prospective including AI and deep learning need further validation to confirm their diagnostic utility and role in the management plans of EC.

In conclusion, the preoperative ADCmean values of EC were significantly correlated with main prognostic factors including depth of MI, CSI, EC type, grade, nodal involvement, and LVSI. Also, there was a statistically significant negative correlation between ADC values with the 2009 FIGO stage and the updated 2023 FIGO stage. Further prospective studies on the correlation between ADC and the updated 2023 FIGO classification system are recommended.

### Electronic supplementary material

Below is the link to the electronic supplementary material.

Supplementary Material 1: figure (1): Bar charts for the agreement between DWI and CE-MRI for depth of MI (a), the agreement between DWI and CE-MRI with the pathological result of the depth of MI (b and c respectively).

## Data Availability

All data generated or analyzed during this study are included in this published article.
