# Supplementary figures and images for: Diagnostic utility of apparent diffusion coefficient in preoperative assessment of endometrial cancer: are we ready for the 2023 FIGO staging?
Source: BMC Med Imaging. 2024 Aug 28;24:226. doi: 10.1186/s12880-024-01391-5 (PMC11351078; doi:10.1186/s12880-024-01391-5)

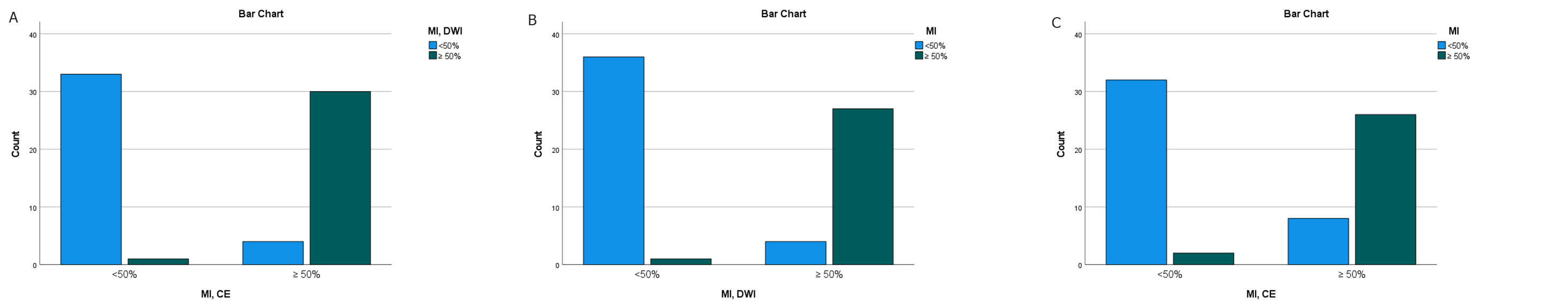

Supplement: Supplementary file 1 — Supplementary Material 1: figure (1): Bar charts for the agreement between DWI and CE-MRI for depth of MI (a), the agreement between DWI and CE-MRI with the pathological result of the depth of MI (b and c respectively). [file 12880_2024_1391_MOESM1_ESM.tif]
